# Expression of *Histophilus somni* IbpA DR2 protective antigen in the diatom *Thalassiosira pseudonana*

**DOI:** 10.1007/s00253-017-8267-8

**Published:** 2017-04-12

**Authors:** Aubrey Davis, Lauren T. Crum, Lynette B. Corbeil, Mark Hildebrand

**Affiliations:** 1 Marine Biology Research Division, Scripps Institution of Oceanography, 9500 Gilman Dr, La Jolla, CA 92093 USA; 2 Department of Pathology, School of Medicine, UC San Diego, San Diego, CA USA

**Keywords:** Diatom, *Thalassiosira pseudonana*, IbpA DR2, *Histophilus somni*, Microalgae, Valuable proteins, Protective antigen

## Abstract

Increasing demand for the low-cost production of valuable proteins has stimulated development of novel expression systems. Many challenges faced by existing technology may be overcome by using unicellular microalgae as an expression platform due to their ability to be cultivated rapidly, inexpensively, and in large scale. Diatoms are a particularly productive type of unicellular algae showing promise as production organisms. Here, we report the development of an expression system in the diatom *Thalassiosira pseudonana* by expressing the protective IbpA DR2 antigen from *Histophilus somni* for the production of a vaccine against bovine respiratory disease. The utilization of diatoms with their typically silicified cell walls permitted development of silicon-responsive transcription elements to induce protein expression. Specifically, we demonstrate that transcription elements from the silicon transporter gene *SIT1* are sufficient to drive high levels of IbpA DR2 expression during silicon limitation and growth arrest. These culture conditions eliminate the flux of cellular resources into cell division processes, yet do not limit protein expression. In addition to improving protein expression levels by molecular manipulations, yield was dramatically increased through cultivation enhancement including elevated light and CO_2_ supplementation. We substantially increased recombinant protein production over starting levels to 1.2% of the total sodium dodecyl sulfate-extractable protein in *T. pseudonana*, which was sufficient to conduct preliminary immunization trials in mice. Mice exposed to 5 μg of diatom-expressed DR2 in whole or sonicated cells (without protein purification) exhibited a modest immune response without the addition of adjuvant.

**Electronic supplementary material:**

The online version of this article (doi:10.1007/s00253-017-8267-8) contains supplementary material, which is available to authorized users.

## Introduction

The ability to produce valuable proteins has been developed in various organisms, including bacteria, yeast, baculovirus, mammalian cells, and plants. Each system has useful traits but also limitations that include the cost of growing expression organisms on rich or highly defined medium, maintenance of sterility, slow generation time, and in some cases a requirement to screen for potential pathogens. Unicellular microalgae are an attractive alternative for protein expression (Mayfield et al. 2007; Rasala et al. 2010; Rasala and Mayfield 2014). Single-celled algae are 10–100 times more productive than plants (Chisti 2007), have rapid generation times, and can be grown photosynthetically in simple medium without additional carbon, which reduces cost and potential contamination issues. Microalgae also exhibit flexibility in growth mode, and certain species can be grown autotrophically, mixotrophically, or heterotrophically, cultivated outdoors, in enclosed photobioreactors, or using fermenter technology, to provide year-round production. Algae can be scaled up quickly from lab-scale cultivation to photobioreactors or acre-sized outdoor ponds, yielding large amounts of cheaper products in a short period of time. Algal-based protein production is already estimated to be similar in cost to the least expensive protein expression systems available (Dove 2002) and much less expensive than mammalian cell culture (Chisti 2007; Rasala et al. 2010). Further reductions in cost, by as much as 10-fold (Rasala et al. 2010), are anticipated through improvements in recombinant protein yield, which has been under-investigated in microalgae, and through improvements in cultivation and harvesting, which is being driven by the development of algae for biofuel and bioproduct production.

The green alga *Chlamydomonas reinhardtii* is the most extensively developed system for recombinant protein production in algae. Variability in expression levels has been documented, ranging from a fraction of a percent to 10% of total soluble protein, which relates to the specific protein being expressed (Rasala and Mayfield 2014). It has also been demonstrated that proteins requiring complex folding, such as antibodies, can be produced successfully with this system (Tran et al. 2009) and that cells can be directed to secrete recombinant proteins into the surrounding medium (Lauersen et al. 2013). Protein expression in *C. reinhardtii* is generally limited to the chloroplast because foreign genes introduced into the nucleus are subjected to silencing, and attempts at nuclear-based expression have resulted in low yields (Jones et al. 2012). Further, proteins expressed in the chloroplast lack post-translational modifications due to the bacterial origin of plastids. However, improvements in nuclear transgene stability and protein expression have been reported using a combined production and secretion approach (Lauersen et al. 2013). Until recently, the technology dictated non-photosynthetic growth by addition of a carbon source (Jones et al. 2012); however, new developments have enabled protein production under autotrophic conditions (Gimpel et al. 2015).

Diatoms are a highly productive class of unicellular eukaryotic microalgae that contribute significantly to the global carbon cycle (Granum et al. 2005). Diatoms generally outcompete other algae in terms of growth (Brzezinski et al. 2001; Carter et al. 2005), and their high productivity indicates an intrinsic efficiency in converting sunlight and CO_2_ to useful products. For example, diatoms are promising candidates for algal-based biofuel production, in which low cost and high productivity are essential (Sheehan et al. 1998; Hildebrand et al. 2012). Diatoms have unique features, including cell walls (referred to as frustules) which are comprised of nanostructured silica. More specifically, the frustule is composed of structures that consist of an amalgam of mesoporous particulate silica 10–100 nm in size (Chiappino and Volcani 1977; Schmid and Schulz 1979; Crawford et al. 2001; Noll et al. 2002). Although it presents a barrier, the cell wall is easily disrupted by detergent treatment or sonication, the former of which disrupts connections between larger silica cell wall structures and the latter of which generates small silica particles. Interestingly, nanoparticulate mesoporous silica has been shown to have efficacy as an adjuvant by boosting the immune response (Carvalho et al. 2010; Mody et al. 2013). We recently demonstrated the adjuvanticity of diatom silica (diatomaceous earth) in an immunization study with Newcastle disease virus in chickens (Nazmi et al. 2016).

A consequence of frustule formation in diatoms is a strict requirement for silicon, the availability of which controls cell cycle progression. Favorable metabolic conditions, related to the availability of carbon, nitrogen, and cellular energy that can be channeled into protein synthesis rather than biomass accumulation, occur during silicon limitation (Darley and Volcani 1969). Silicon metabolism is generally uncoupled from nitrogen metabolism (Claquin et al. 2002); thus, silicon starvation would be expected to have little effect on protein synthesis potential. In the most highly developed recombinant protein expression systems, such as bacteria and yeasts, conditions in which cellular growth is blocked or severely retarded are desirable because they permit surplus energy and metabolic potential to flow into recombinant protein expression (Dove 2002; Jonasson et al. 2002).

Diatoms offer additional advantages relative to some types of algae because nuclear transformation in diatoms is not affected by transgene silencing (Dunahay et al. 1995; Hempel et al. 2011), and the expression of genes in the nucleus offers the possibility of post-translational protein modifications and targeting to multiple intracellular locations. Further, the ease with which the walls of silicified diatoms are disrupted translates into reduced effort to extract or expose expressed proteins. In the case of vaccines, antigencity can be substantially improved in conjunction with nanoparticulate or microparticulate materials (O’Hagan and Singh 2014) suggesting that either intact or sonicated silicified diatoms could be used without the need to purify the expressed antigen.

The non-silicified diatom *Phaeodactylum tricornutum* has been used to express human IgG antibody against the hepatitis B surface protein, plus the antigen itself (Hempel et al. 2011). Yields were 8.7% of total soluble protein for the antibody and 0.7% for the antigen (Hempel et al. 2011). Such variability in yield, depending on the protein expressed, is not uncommon (Rasala and Mayfield 2014). Fully functional IgG was also expressed and secreted into the growth medium at concentrations up to 2.5 μg ml^−1^ (Hempel and Maier 2012). Because *P. tricornutum* does not require silicon for biomass accumulation, it is not possible to leverage silicon limitation as a strategy for channeling cellular resources away from ancillary processes and into protein synthesis.

Genetically engineered biosilica purified from the silicified diatom *Thalassiosira pseudonana* has shown promise as a drug delivery system (Delalat et al. 2015). In contrast, we are interested in developing diatoms with silicified cell walls as an intact package for vaccine production and delivery systems (Corbeil et al. 2015). There are some advantages to consider, particularly in the application of this technology to developing areas of the world. Diatoms can be grown inexpensively in a variety of water sources at low cost; lyophilization of cultivated diatoms could eliminate the need for cold temperature storage, and diatom-expressed proteins could provide an all-in-one package of vaccine and adjuvant since diatom silica has been shown to enhance immune response (Nazmi et al. 2016). As a test case, we decided to express the IbpA DR2 antigen from *Histophilus somni* for vaccine development against bovine respiratory disease. We previously demonstrated that this purified recombinant subunit antigen produced in *Escherichia coli* protects against *H. somni* pneumonia in calves and septicemia in mice (Geertsema et al. 2008; Geertsema et al. 2011). In this report, we adopted a stepwise approach to examine and evaluate a variety of parameters affecting recombinant IbpA DR2 antigen expression in *T. pseudonana*, with a goal of achieving expression levels that would elicit an immune response in mice.

## Materials and methods

### Diatom cultivation

*T. pseudonana* (Bigelow Laboratory for Ocean Sciences NCMA strain CCMP 1335) was cultivated in artificial seawater (ASW) medium (Darley and Volcani 1969) at 18 °C under continuous light. Depending on experimental design, light conditions ranged from 180 to 500 μmol photons m^−2^ s^−1^, as measured using an Apogee MQ-200 light meter. Transformant lines constitutively expressing IbpA DR2 were grown in 8-L batches to a density of ∼1 × 10^6^ cells ml^−1^ and then imaged or harvested for protein extraction or mouse immunizations. Transformant lines utilizing an inducible, silicon-responsive gene (SIT1) promoter were initially grown in 3 L starter cultures in replete ASW medium to a density of ∼1 × 10^6^ cells ml^−1^ and then harvested by filtration on 3-μm TSTP Isopore filters (EMD-Millipore, Billerica, MA, USA). The equivalent of 6 L of material harvested from starter cultures was resuspended in 8 L of ASW without silicon for 24 h. Cell density was determined to ensure cessation of growth in silicon-free medium, and cells were harvested by filtration as described above, rinsed with 0.5 M mannitol to remove salts while maintaining cell integrity, pelleted by centrifugation, and frozen at −80 °C. Cell counts were performed with the Muse Cell Analyzer (EMD-Millipore, Billerica, MA, USA).

### Vector construction for IbpA DR2 expression

The direct repeat 2 Fic domain (DR2/Fic, hence called DR2) of the immunoglobulin-binding protein A (IbpA) from *H. somni* (accession number AB087258.1) was codon optimized (Supplementary Fig. S2) for diatom expression based on the Kasuza codon usage database (http://www.kazusa.or.jp/codon/cgi-bin/showcodon.cgi?species=35128) and synthesized by Integrated DNA Technologies, Inc. (San Diego, CA). To prevent possible cytotoxic effects from inhibiting expression in *T. pseudonana*, we inactivated the Fic domain by substituting His for Ala (H:A) as demonstrated by Worby et al. (2009), with the QuikChange Lightning Site-Directed Mutagenesis Kit (Agilent Technologies, Santa Clara, CA) following the manufacturer’s suggestions. Accession numbers for the codon optimized and H:A sequences are KY582960 and KY582961, respectively. Expression differences between Fic domain-containing constructs, IbpA DR2-GFP, and mutagenized constructs, IbpA DR2 H:A-GFP, were examined.

Entry vectors and destination vectors intended for expression were constructed as described by Shrestha and Hildebrand (2015) using MultiSite Gateway Technology (Thermo Fisher Scientific, Waltham, MA, USA). Briefly, the IbpA DR2 domain was PCR amplified and cloned into the Gateway donor vector pDONR221 attP1–attP2 to generate an entry vector. The constitutive expression vector pTpfcpGFP (Poulsen et al. 2006) was modified by cloning a Gateway frame B cassette upstream of e*gfp* to create a destination vector (pMHL_79) driven by *fcp* regulatory elements. The destination vector constructed with the regulatory elements that drive inducible expression of the silicon transporter gene *SIT1* (Thaps3_268893) in silicon-limiting conditions (Shrestha and Hildebrand 2015; Smith et al. 2016) was synthesized by GeneArt Gene Synthesis (Thermo Fisher Scientific, Waltham, MA, USA) and consisted of the Gateway frame B cassette nested between a 429 bp upstream region and a 505 bp downstream region of the *SIT1* gene cloned into the pMA vector. This destination vector was further modified by cloning *bfloGFPa1* (Bomati et al. 2009) downstream of the Gateway frame B cassette generating the inducible expression vector pMHL_2002. IbpA DR2-GFP, IbpA DR2 H:A-GFP, and chloroplast-targeted IbpA DR2 H:A-GFP-inducible expression constructs were created by cloning PCR fragments into appropriate entry vectors followed by recombination into the pMHL_2002 destination vector with MultiSite Gateway Technology following the manufacturer’s recommendations. Gene-specific primer sequence used to amplify fragments for construct synthesis are listed in Supplementary Table S1. Each expression vector was co-transformed with pMHL_9 expressing the *nat1* gene, which confers resistance to the antibiotic nourseothricin, under the control of the acetyl coenzyme A carboxylase promoter.

### Diatom transformation

The protocol for diatom transformation was adapted from Poulsen et al. (2006). Axenic exponential-phase wild-type *T. pseudonana* cells (1 × 10^8^ cells total) were pelleted (3000*×g* for 10 min) and plated on ASW medium 1.2% agar plates. M-17 tungsten particles were coated with 5 μg of plasmid DNA for each construct with the CaCl_2_-spermidine method as per the manufacturer’s instructions (Bio-Rad, 165-2267). Each plate was bombarded twice with 2–5 mg of coated tungsten beads using the Biolistic DS-1000/He particle delivery system with a 1350 psi rupture disk. Following bombardment, cells were immediately resuspended in 10 ml of ASW medium and incubated for 24 h under constant illumination (150 μE m^−2^ s^−1^). The following day, the cell density was determined and a range of concentrations (1 × 10^6^–5 × 10^6^ cells) were plated on ASW agar plates containing the antibiotic nourseothricin at a final concentration of 100 μg ml^−1^. The plates were incubated at 18 °C in continuous light for approximately 2 weeks. Individual colonies from the plates were isolated and screened by PCR and fluorescence microscopy to confirm nuclear insertion of the plasmid and for GFP expression, respectively.

### Fluorescence microscopy

Diatoms were imaged with a Zeiss Axio Observer Z1 inverted microscope equipped with an ApoTome and a Zeiss AxioCam MRm camera (Carl Zeiss Microimaging, Inc., Thornwood, NY, USA). Non-fluorescent images were taken using differential interference contrast (DIC). The filter sets used for fluorescent imaging were as follows: chlorophyll (Zeiss #16 excitation BP 485/20 nm, dichromatic mirror FT 510 nm, emission LP 515 nm) and GFP (Zeiss #38HE excitation BP 470/40 nm, dichromatic mirror FT 495 nm, emission BP 525/50 nm). Images were acquired with a ×40/0.75 or ×63/1.4 objective oil immersion Plan-Apochromat objective and processed using Axiovision 4.7.2 software.

### Imaging cytometry analysis

*T. pseudonana* cells were analyzed with the ImageStream X (Amnis Corporation, Seattle, WA) imaging flow cytometer. Ten thousand events were acquired for each sample using the INSPIRE software package (Amnis Corporation, Seattle, WA). Post-acquisition analysis was performed using the IDEAS software package provided by the manufacturer (Amnis Corporation, Seattle, WA). “No GFP” and “Background” gates were determined based on wild-type cells lacking GFP fluorescence.

### Western blotting for DR2 expression quantitation

Diatoms expressing IbpA DR2-GFP or IbpA DR2 H:A-GFP were harvested by centrifugation and preserved as cell pellets at −20 °C until processed. Cell lysis buffer (2% sodium dodecyl sulfate (SDS), 62.5 mM Tris, pH 6.8) was added to the pellets at 5 volumes of wet weight and boiled for 5 min to yield total SDS-extractable protein. Samples were centrifuged, and the supernatant was retained. Protein concentration in the supernatant was determined by using a DC protein assay (Bio-Rad, Hercules, CA), and 5 μg of total protein was loaded per well for each sample. A standard curve was made from recombinant IbpA DR2 by serially diluting untagged rDR2 protein (kindly provided by J. Xiao and C Worby) (Xiao et al. 2010). Samples and standards were boiled in SDS sample buffer and dithiothreitol (DTT) and loaded on 10% polyacrylamide gels. After SDS-PAGE electrophoresis, proteins were transferred to a nitrocellulose membrane (0.45 μm, Bio-Rad, made in Germany) at 30 V overnight followed by 70 V for 1 h. After blocking for 1 h with 0.3% gelatin in Tris-buffered saline (TBS)/0.05% Tween 20, blots were incubated with rabbit 405 anti-rIbpA DR2 antibody (Zekarias et al. 2010) at a dilution of 1:1000 for 2 h. After washing three times, blots were incubated with alkaline phosphatase conjugated goat anti-mouse IgG (Zymed) at 1:8000 for 1 h. Blots were washed three times and developed with One-Step NBT/BCIP (nitroblue tetrazolium/5-bromo-4-chloro-3-indoly phosphate, Thermo Scientific, Rockford, IL). Densitometry was performed using ImageJ software. A standard curve based on untagged rDR2 (Xiao et al. 2010) was used to quantitate the amount of IbpA DR2-GFP or DR2 H:A-GFP expressed in the diatoms.

### Mouse immunizations

Female, 5–6 weeks old, Swiss Webster mice obtained from Charles River Labs were housed in groups of four in ventilated cages. Immunization experiments were conducted in two separate trials: first, a dose response experiment to determine the amount of antigen necessary for protection, then a study using the antigen expressing diatoms. In each experiment, four animals per group were immunized subcutaneously with antigens and adjuvant three times at 3-week intervals (see below for composition of inocula). Blood was drawn from representative mice at time 0 and from each mouse 2 weeks after each immunization. Approximately 50 μl of blood was collected by mandibular puncture 2 weeks after the first and second immunization. Blood was collected by cardiac puncture after euthanasia 2 weeks after third immunization. Serum was stored at −20 °C until antibody analysis.

#### Dose response study with recombinant *H. somni* IbpA DR2 protein in PBS or Quil A adjuvant

Mice were immunized with 0.4, 2, and 10 μg of the rDR2 purified from *E. coli* as previously described (Geertsema et al. 2011). Recombinant proteins were diluted in phosphate-buffered saline (PBS), pH 8.0, to 100 μl, then mixed with either 100 μl PBS or 10 μg Quil A (Accurate Chemical and Scientific, Westbury, NY) in 100 μl PBS before being injected subcutaneously in the mice. Three and 6 weeks later, all mice received a second and third immunization. Antibody responses were determined by ELISA, as described below.

#### Immune response to diatom-expressed *H. somni* IbpA DR2-GFP or IbpA DR2 H:A-GFP

Mice were immunized with sonicated diatoms expressing 5 μg IbpA DR2-GFP, sonicated diatoms expressing 5 μg IbpA DR2 H:A-GFP, or whole diatoms expressing 5 μg IbpA DR2 H:A-GFP. Sonicated diatoms were treated on ice with a Fisher Sonic Dismembrator model 300 at a setting of 30 for three pulses at 10 s each with a 10-s pause in between to allow sample to cool. Mice were immunized subcutaneously with diatoms in 200 μl of PBS. Three and 6 weeks later, all mice received a second and third immunization.

All animal experiments were approved by the University of California San Diego Institutional Animal Care and Use Committee.

### ELISA of immunized mouse serum

Specific serum IgG antibodies were analyzed against *H. somni* rDR2 antigen by ELISA as previously reported (Geertsema et al. 2008). Briefly, 96-well plates (Costar-High Binding EIA/RIA one-half well plates, Corning Inc., Corning, NY) were coated overnight at room temperature with rDR2 (purified from *E. coli*) at 100 ng/well. After blocking overnight with 3% porcine gelatin in PBS with 0.02% sodium azide, wells were incubated for 1.5 h in a humid chamber at 37 °C. Mouse serum from the dose response experiment was diluted to 1:1000. Serum from the diatom-expressed antigen immunization was diluted to 1:100. Positive and negative controls consisted of convalescent phase mouse serum from a previous study (Geertsema et al. 2008a) and naïve mouse serum, respectively. Each serum was tested in duplicate. Plates were washed three times with PBS/0.05% Tween 20, and antibody level was detected by incubating for 1 h with horseradish peroxidase-conjugated goat anti-mouse IgG (Invitrogen, Camarillo, CA) at 1:2000 followed by tetramethylbenzidine/hydrogen peroxide (TMB) substrate (KPL, Gaithersburg, MD). After 15 min, the reaction was stopped with 1 N HCl and absorbance was determined by reading at A450/A650 in a dual-wavelength microplate reader (Molecular Devices Corp., Menlo Park, CA). Data was reported as absorbance values ± the standard errors.

## Results

### Development of inducible expression for enhanced protein production

Development of a diatom-based expression system requires the identification of promoters with the ability to drive the production of high levels of recombinant protein. We have developed a promoter system using the *T. pseudonana* silicon transporter *SIT1* gene (Thamatrakoln and Hildebrand 2007; Shrestha and Hildebrand 2015), which is highly induced during silicon limitation and repressed during growth in silicon replete conditions (Shrestha and Hildebrand 2015). Upstream and downstream flanking sequences of *SIT1* were shown to be sufficient for high level and inducible expression under silicon limitation (Shrestha and Hildebrand 2015).

The *fcp* promoter has been routinely used in transgenic diatom studies (Apt et al. 1996; Falciatore et al. 1999; Poulsen and Kröger 2005; Poulsen et al. 2006; Siaut et al. 2007; Miyagawa et al. 2009; Miyagawa-Yamaguchi et al. 2011) to drive constitutive expression. Assuming the premise that constitutive recombinant protein expression could induce a metabolic drain on the cell, we generated two constructs for IbpA DR2-GFP expression: one driven by the constitutively expressed *fcp* control elements and the other by the inducible *SIT1* elements (Supplemenatry Fig. S1).

**Fig. 1:**
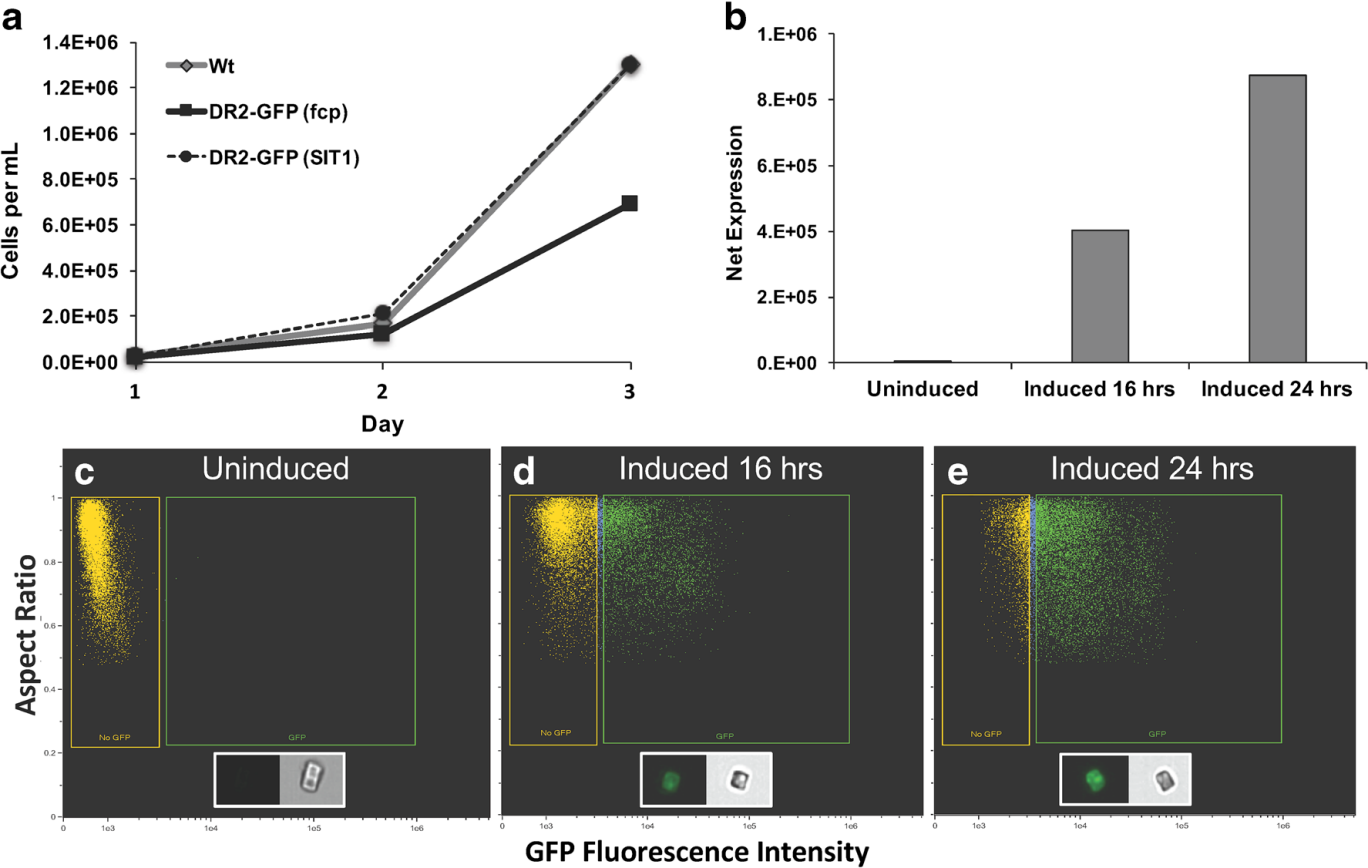
Growth and expression characteristics of IbpA DR2-GFP transformed lines of *T. pseudonana*. **a** A representative experiment demonstrating growth differences between wild-type cells and cells expressing IbpA DR2-GFP under the control of either *fcp* or *SIT1* regulatory elements in silicon-replete medium. *Wt* wild type, *DR2-GFP (fcp) fcp*-driven expression, *DR2-GFP (SIT1) SIT1*-driven expression (cells not induced). **b** Net expression of pTp SIT1/IbpA DR2-GFP as determined by imaging flow cytometry. Net expression calculated as the mean GFP fluorescence intensity in the population of GFP-gated cells multiplied by the percentage of the total population of cells expressing GFP. Expression differences between cells growing in replete medium (uninduced) and medium without silicon (induced) can be observed. **c**–**e** Scatterplots demonstrating the effect of silicon availability on the expression of pTp SIT1/IbpA DR2-GFP as observed with imaging flow cytometery. **c** No detectable expression based on GFP fluorescence under silicon replete conditions. **d** Expression after 16 h of silicon starvation. **e** Expression after 24 h of silicon starvation. *Yellow* and *green gates* represent no GFP and GFP fluorescence, respectively. Gates were determined based on wild-type cells without GFP. Ten thousand cells were analyzed for each sample

An examination of growth comparing the two expression approaches indicated that the *fcp*-driven construct reduced growth in this particular experiment (1.9-fold less) by day 3 as compared to the similar responses exhibited by wild-type cells and cells carrying the uninduced *SIT1*-driven construct (Fig. 1a). This could potentially have been due to a detrimental effect specifically related to IbpA DR2-GFP or to a metabolic and energetic drain on cells expressing excessive levels of a protein during growth. In either case, the use of an inducible promoter is beneficial in that more abundant biomass can be accumulated prior to initiating protein expression. To test the inducibility of the *SIT1*-driven construct, we compared *SIT1* expression before (uninduced) and after (induced) cultivation in silicon-free medium for 16 or 24 h (Fig. 1b). The comparison was made based on net expression—a proxy for the total amount of protein yield per culture volume, which was determined by multiplying the mean GFP fluorescence intensity of the population of cells captured with imaging flow cytometry by the percentage of cells in the population with detectable GFP fluorescence (Fig. 1c–e). The data show that *SIT1*-driven expression was minimized during growth in abundant silicon but induced by over 50-fold after 24-h silicon starvation (Fig. 1).

### Assessing IbpA DR2-GFP expression in *T. pseudonana*

**Fig. 2:**
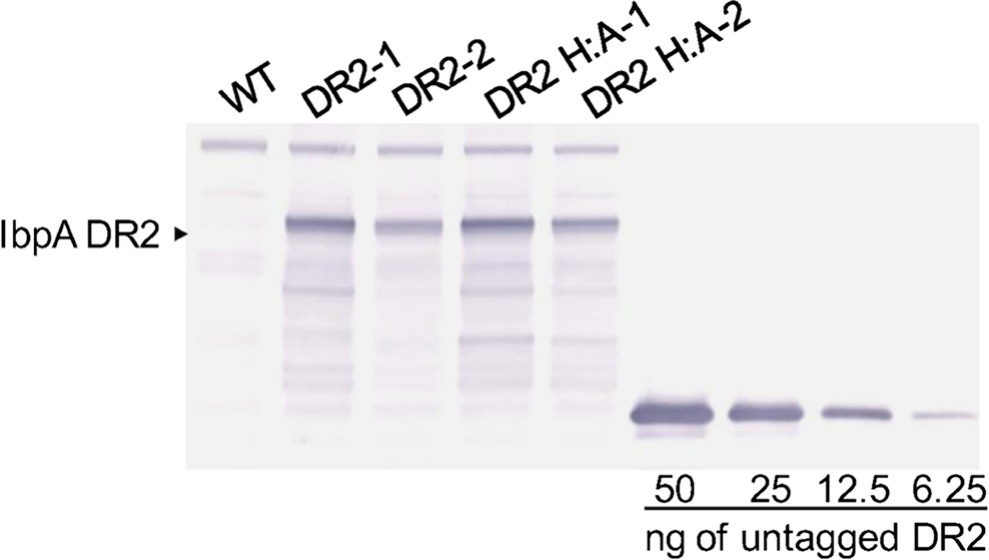
Quantitation of IbpA DR2 expression in diatoms by Western analysis. *Wt* wild-type diatoms, *DR2-1* and *DR2-2* batches 1 and 2 of IbpA DR2 expressing diatoms, *DR2 H:A-1* and *DR2 H:A-2* batches 1 and 2 of diatoms expressing IbpA DR2 with the critical histidine in the catalytic site replaced by alanine. Quantitation was based on densitometric comparisons with known amounts of DR2 protein

Fusions with GFP allowed detailed characterization of IbpA DR2 expression within individual cells and rapid assessment of expression within populations of cells. However, strict quantitation of IbpA DR2 expression levels in *T. pseudonana* was performed by Western analysis in combination with densitometry. We utilized an IbpA DR2-specific antibody (Zekarias et al. 2010) against a titration of recombinant IbpA DR2 produced in *E. coli* (rDR2) (Xiao et al. 2010). The DR2-GFP fusion produced in *T. pseudonana* was readily detected (Fig. 2) and permitted quantitation through densitometric comparison with purified untagged IbpA rDR2 (Xiao et al. 2010). These methods facilitated stepwise assessment and improvement of the expression system. Further, quantitation of IbpA DR2 was necessary in order to determine doses for murine immunization studies. For this reason, we monitored total extractable protein (SDS-extractable) rather than total soluble protein because our desired delivery mechanism was whole or sonicated diatom cells, not purified protein (see “Discussion” section).

### The Fic domain and expression in diatoms

**Fig. 3:**
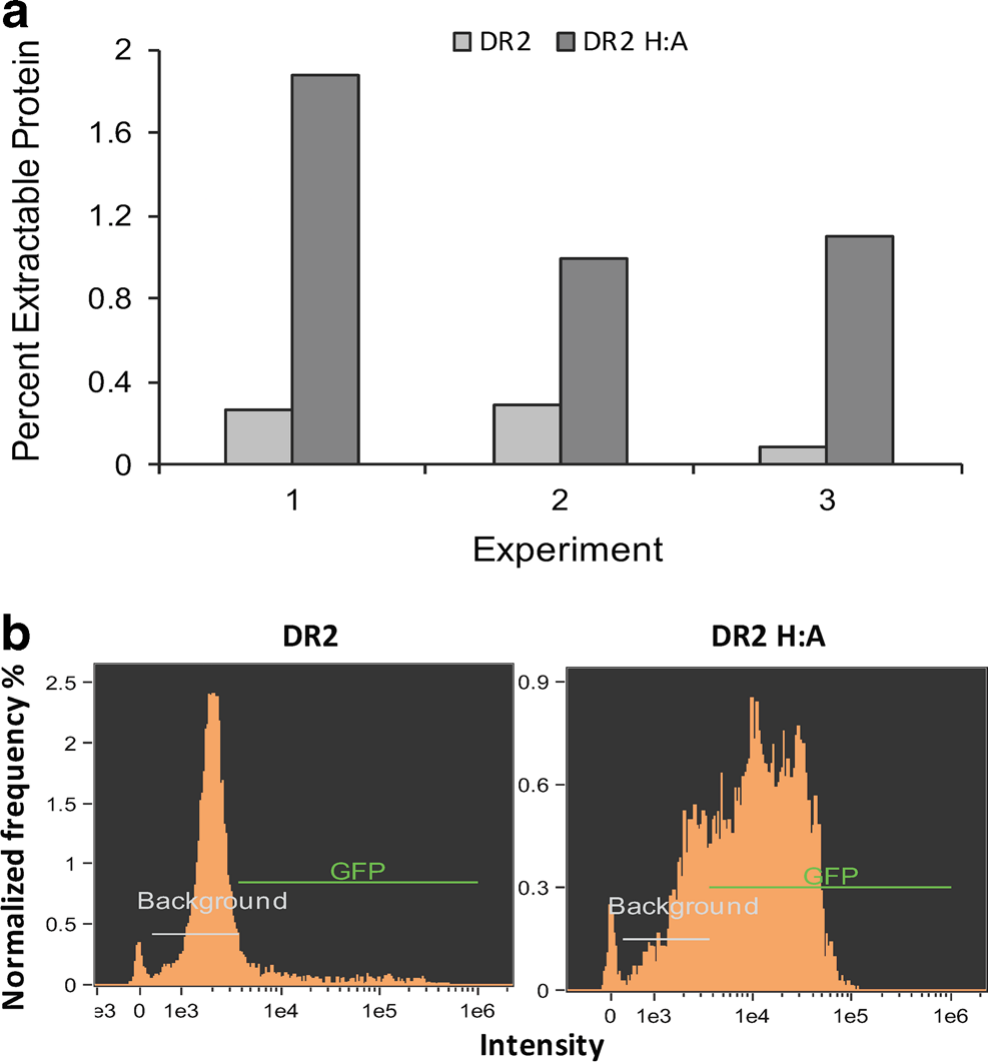
Comparison of IbpA DR2 expression with and without an active Fic domain. **a** Comparison of percent extractable protein from cells expressing the native IbpA DR2 (pTp SIT1/IbpA DR2-GFP) and cells expressing the mutagenized DR2 without an active Fic domain (pTp SIT1/IbpA DR2 H:A-GFP) during three experiments. Percent extractable protein was determined by Western analysis. **b** Imaging cytometry analysis of the frequency of cells vs. intensity of GFP fluorescence in a representative culture. Expression improvements occurring in the population expressing DR2 H:A-GFP, as compared to populations expressing DR2-GFP with an active Fic domain, arise from an increase in both the percent of the population expressing as well as an increase in expression per cell

The native IbpA DR2 protein contains a cytotoxic Fic domain, which catalyzes the addition of AMP to the switch I region of Rho GTPases, preventing interaction with downstream effectors and resulting in cytoskeletal disruption (Worby et al. 2009). Cytotoxicity of the conserved Fic motif (HPFXXGNGR) depends on a catalytic His residue (Zekarias et al. 2010). To avoid any potentially negative effects on expression levels, we generated a construct in which the histidine was converted to an alanine (Ibpa DR2 H:A), rendering the Fic domain inactive (Worby et al. 2009). Western analysis using the IbpA DR2-specific antibody revealed that the IbpA DR2 H:A-GFP construct resulted, on average, in a 6.1-fold increase (*n* = 3) in expressed protein after induction as compared to IbpA DR2-GFP construct when cells were cultivated under similar conditions (Fig. 3a). In conjunction with bulk analysis of expression through Western blotting methods, imaging cytometry revealed details of expression differences between populations transformed with the two constructs. Higher net expression levels observed with IbpA DR2 H:A-GFP were driven largely by a dramatic increase in the percent of cells in the population expressing rather than an increase in expression levels per cell. In a representative experiment, only 11% of IbpA DR2-GFP transgenic cells expressed GFP, whereas 75% expressed GFP in IbpA DR2 H:A-GFP transgenic cells (Fig. 3b). These results suggest that cells were experiencing some level of toxicity and demonstrate that IbpA DR2 yield was improved by Fic domain inactivation.

### Targeting expressed protein to multiple cellular compartments to increase overall yield

**Fig. 4:**
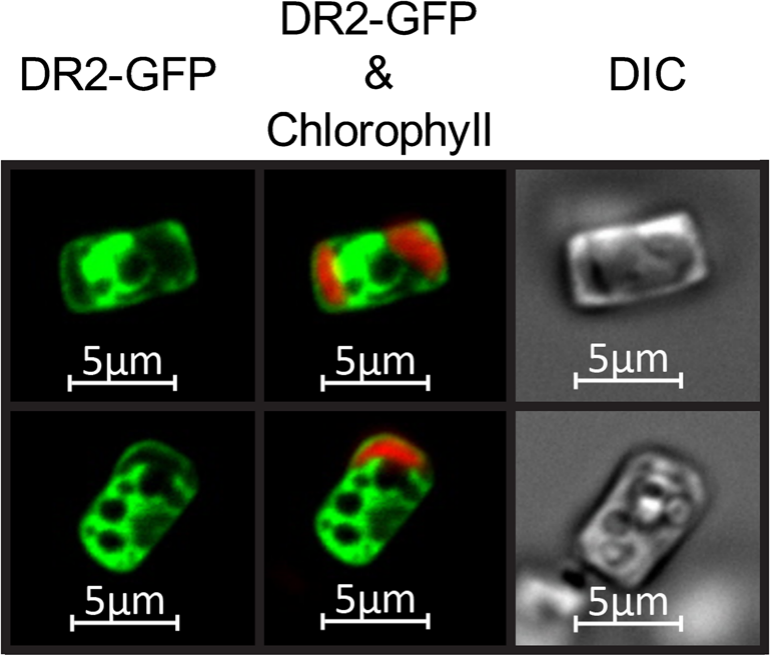
Fluorescence imaging of IbpA DR2-GFP expressed in *T. pseudonana*. Cytoplasmic distribution of IbpA DR2-GFP under the control of *SIT1* expression elements during induction. Chlorophyll fluorescence shown in *red*; GFP shown in *green*; dark areas are other cellular compartments

**Fig. 5:**
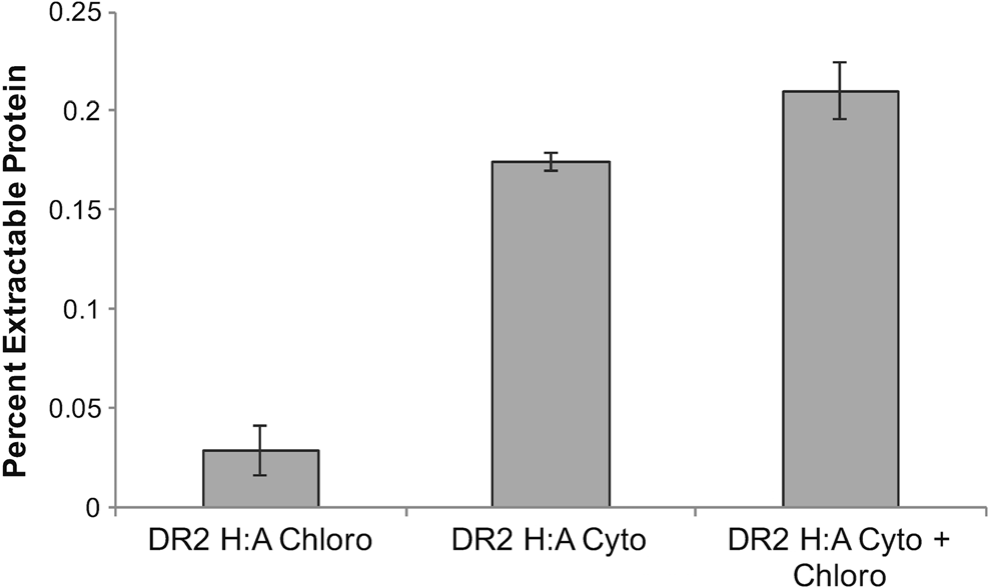
Comparison of IbpA DR2 H:A-GFP protein expression levels with chloroplast targeted, cytoplasmic targeted, and combined chloroplast and cytoplasmic targeted transgenic lines. Mean values of Western analysis of IbpA DR2 H:A-GFP expressed as a percent of the total extractable protein. Data are from replicate experiments (*n* = 2)

Fluorescence microscopy of individual *SIT1*-induced IbpA DR2-GFP expressing cells, in which the protein was cytoplasmically targeted, revealed that the entire cytoplasm contained GFP fluorescence (Fig. 4). Non-cytoplasmic compartments, such as vacuoles, appear as dark areas lacking fluorescence surrounded by cytoplasmic labeling (Fig. 4). These observations led us to evaluate whether simultaneously targeting expressed protein to multiple cellular locations would increase yield by taking advantage of additional cellular volume. Because chloroplasts occupy a significant portion of non-cytoplasmic space and targeting signals have been well characterized in diatoms (Apt 2002; Gruber et al. 2007), we selected this organelle as a test case to evaluate whether targeting of expressed protein to additional cellular compartments would increase overall yield. Transgenic lines were generated expressing IbpA DR2 H:A-GFP in the cytoplasm only, in the chloroplast only, and in both the cytoplasm and chloroplast. Western analysis on replicate culture experiments showed that chloroplast expression was 16.5% of the level of cytoplasmic expression and that combined cytoplasmic and chloroplast expression increased yield by 17% (Fig. 5). Although the dually targeted lines yielded more expressed protein than the cytoplasmically targeted lines, the percentage increase was slight, and we noticed that over cultivation passages, there was a decrease in chloroplast-targeted expression. It is likely that continued selection for high GFP fluorescence could be applied to maintain chloroplast-targeted expression; however, this was not pursued in subsequent experiments due to the small improvement in overall protein yields.

### Improved protein yield through cultivation optimization

**Fig. 6:**
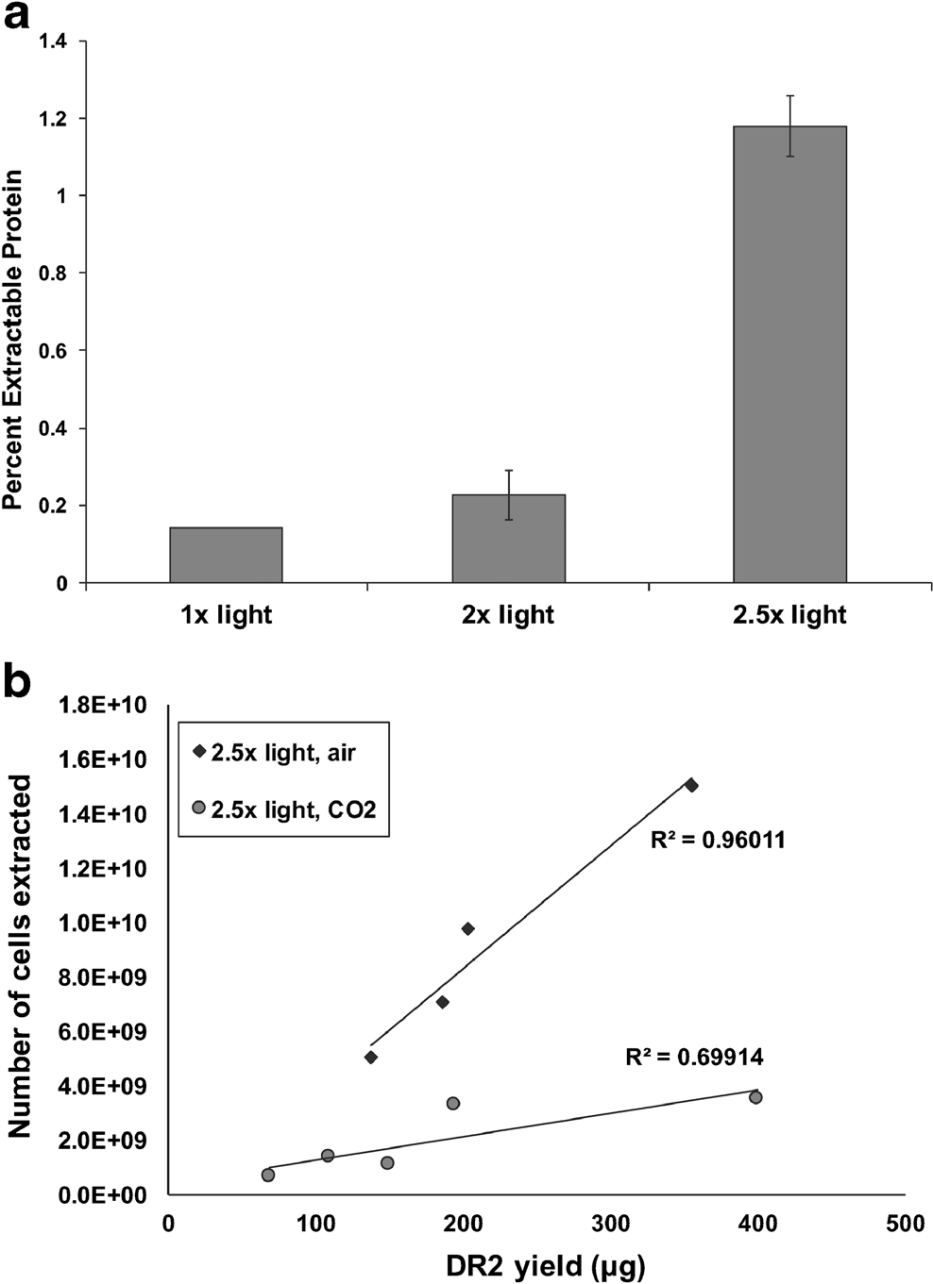
The effect of light intensity and CO_2_ on IbpA DR2 H:A-GFP expression and culture yield. **a** Effect of increasing light intensity on DR2 expression. **b** Data from several cultures were pooled and plotted. Values are normalized according to culture density at 1 × 10^9^ cells per liter. Cultures supplemented with CO_2_ yielded more target protein per cell than cultures grown with air only

After implementation of the stepwise improvements discussed above, maximum expression levels of 0.2% of total extractable protein were achieved, which was far lower than our desired target of 1%. To further develop the system, we explored the effects of increased light intensity and CO_2_ supplementation on protein yield. Standard conditions using fluorescent bulbs produced an intensity of 180 μmol photons m^−2^ s^−1^. With the addition of LED lights with a similar wavelength spectrum, incremental increases in light intensity to 2.5-fold higher were possible. Although improvements were non-linear, increases in intensity significantly enhanced protein expression, resulting in the production of 1.2% of total extractable protein (Fig. 6a). Similarly, the use of smaller diameter cultivation flasks that reduced the light pathlength by half improved expression 3-fold (data not shown). Supplementation with CO_2_ further improved recombinant protein yield. Figure 6b shows the yield of IbpA DR2-GFP per amount of cells harvested from various experiments conducted in similar light conditions but bubbled with either air or 1% CO_2_. In all of the experiments, more recombinant protein was obtained per cell with CO_2_ supplementation, improving protein yield per culture volume. These improvements reduced cultivation effort necessary for the production of the desired protein.

### Antibody responses of mice immunized with diatom-expressed IbpA DR2-GFP

**Fig. 7:**
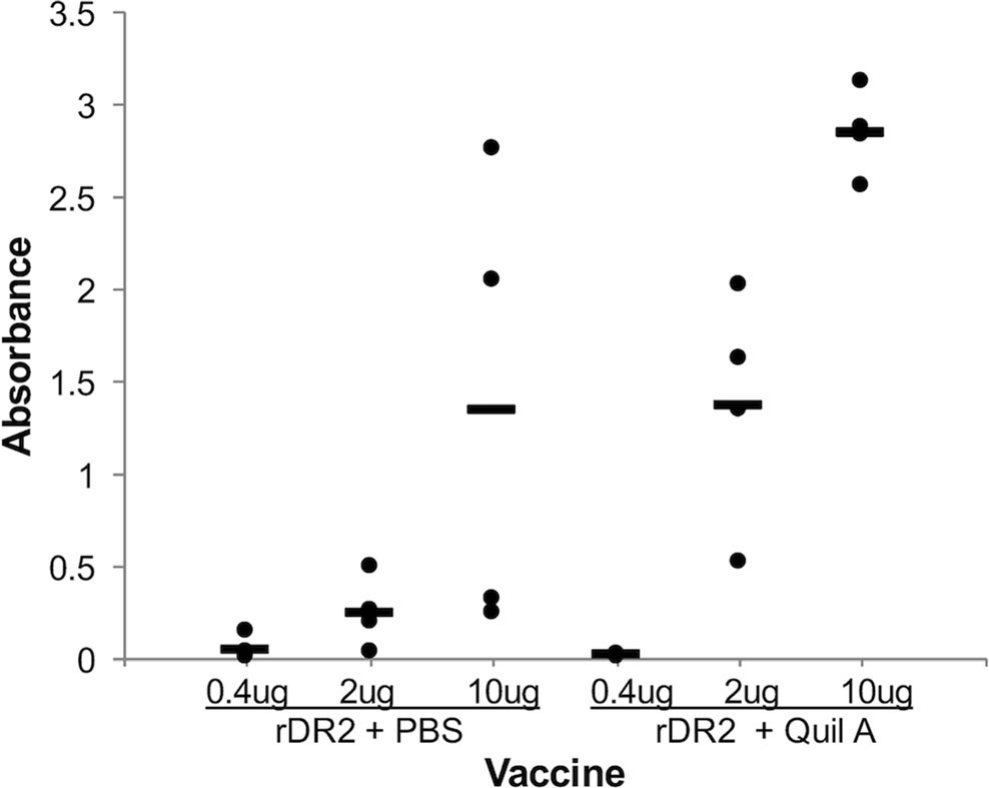
Dose response of mice immunized subcutaneously with purified rDR2 in PBS or Quil A. Serum IgG antibodies to rDR2 measured by ELISA. Each *circle* represents an individual mouse with the mean displayed as a *bar*

**Fig. 8:**
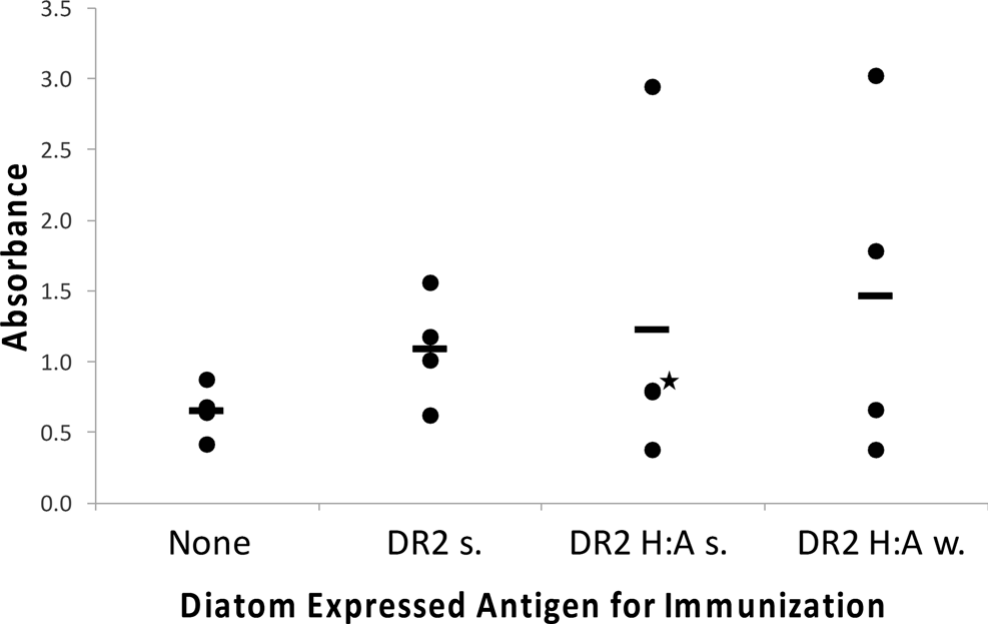
Serum IgG antibody response to rDR2 after the third immunization with diatoms expressing IbpA DR2 or IbpA DR2 H:A-GFP (*s* sonicated diatoms, *w* whole diatoms). Each *circle* represents an individual mouse with the mean displayed as a *bar*. Two circles overlap as indicated by a *star* in the IbpA DR2 H:A-GFP (s) group. Wild-type diatom controls were not included in this experiment because a prior experiment showed that they did not induce a cross-reactive antibody response against IbpA DR2

To determine an effective dose of IbpA DR2 to elicit an IgG antibody response, we injected mice with different amounts of recombinant GST-DR2 fusion protein (rDR2) produced in *E. coli*, with and without Quil A adjuvant (Fig. 7). The data indicate that even without adjuvant at 10 μg of injected protein, two out of four mice demonstrated a response. With the addition of Quil A, all mice responded to 10 μg injected protein, and three out of four mice responded to 2 μg injected protein. An intermediate concentration of 5 μg was selected to evaluate the response to diatom-expressed protein without the addition of adjuvant. Evaluation of IgG serum antibody levels after immunizing mice with an appropriate amount of diatoms to expose them to 5 μg of diatom-expressed IbpA DR2-GFP or IbpA DR2 H:A-GFP suggested a modest immune response relative to controls (Fig. 8). Additionally, the IbpA DR2 H:A-GFP (w) treatment produced a similar response to sonicated diatoms expressing IbpA DR2-GFP or IbpA DR2 H:A-GFP, with two out of four mice responding relative to controls. These results demonstrate that in treatments without the addition of adjuvant, the diatom-expressed mutant IbpA DR2 H:A-GFP was as effective at stimulating an immune response in mice as the diatom-expressed IbpA DR2-GFP.

## Discussion

Here, we report the development of a protein expression system in the silicified diatom *T. pseudonana* through the production of IbpA DR2 from *H. somni*. We have taken advantage of conditions (Si limitation) that eliminate carbon and energy flux into cell division processes, yet do not limit protein expression (Darley and Volcani 1969). The advantages of using inducible promoters to circumvent growth inhibition due to metabolic drain or to the expression of toxic proteins have been demonstrated in expression systems developed in other organisms (Jonasson et al. 2002; Egelkrout et al. 2012). Promoters induced during growth arrest are particularly useful in that more cellular resources are available to be channeled into recombinant protein production due to the reduction of energy and metabolite flux to ancillary processes. The nitrate reductase promoter has been developed as an inducible expression system in diatoms (Poulsen and Kröger 2005; Poulsen et al. 2006); however, induction requires the provision of nitrate, a condition which is not consistent with growth arrest, or nitrogen limitation, which may have negative implications for many cellular processes, including protein synthesis (Martin et al. 1976).

The use of a silicon-responsive promoter to drive expression permits repression under silicon replete conditions and induction under silicon starvation-induced cell cycle arrest and enables the maintenance of toxic proteins. Reduced growth rate in cells constitutively expressing IbpA DR2-GFP (Fig. 1a) and a substantial reduction in the percentage of cells expressing IbpA DR2-GFP relative to IbpA DR2 H:A-GFP (Fig. 3) suggest that cells were experiencing cytotoxicity due to the expression of IbpA DR2-GFP. The Fic domain in IbpA DR2 has been shown to interfere with GTPase signaling pathways (Worby et al. 2009) and may inhibit transcription or protein synthesis in *T. pseudonana*. Silicon replete conditions prevented IbpA DR2-GFP expression under the control of the SIT1 promoter and facilitated rapid biomass accumulation (Fig. 1).

One advantage of nuclear-based expression is the ability to target desired proteins to different cellular compartments. We explored this methodology in *T. pseudonana* by directing IbpA DR2-GFP to the chloroplast. Simultaneous targeting to the cytoplasm and chloroplasts improved yield over cytoplasmic expression alone (Fig. 5). To our knowledge, this is the first demonstration in algae of enhanced recombinant protein yield through simultaneous targeting to more than one cellular compartment. Although gains in expression level were modest in our experiments, several factors could lead to a more substantial contribution. Other diatom species have more chloroplasts per cell than *T. pseudonana*, which would provide greater targeted volume, and other compartments such as the vacuoles occupy a large volume (Fig. 4). Vacuolar-targeting signals have recently been identified in diatoms (Huang et al. 2016) opening the possibility of targeting expressed proteins to that compartment. Subcellular compartment targeting could be taken advantage of in other ways, for example, it has been established in plants that targeting recombinant proteins to the endoplasmic reticulum substantially improves yield due to enhanced protein folding and stability (Conrad and Fiedler 1998; Faye et al. 2005).

Factors beyond the molecular level had dramatic effects on protein yield with substantial gains obtained through the manipulation of various cultivation parameters. Implementation of the molecular improvements discussed above enabled expression levels of ∼0.2% of total extractable protein to be achieved. While this value is similar to maximum expression levels (0.2–0.25%) of heterologous genes expressed in the nuclear genome of *C. reinhardtii* (Neupert et al. 2009; Rasala and Mayfield 2014), considerable improvement was necessary to boost protein yield for downstream applications. While it is intuitive that increased light and CO_2_ availability facilitate protein production in phototrophic conditions, the improvements resulting from these changes were substantial. Increasing light levels by 2.5-fold resulted in a 6-fold increase in expression levels to 1.2% of total extractable protein (Fig. 6a), and CO_2_ supplementation increased protein yield per cell (Fig. 6b). The correlation between recombinant protein expression and light intensity in phototrophic expression systems is consistent with recent observations in *C. reinhardtii* (Gimpel et al. 2015).

Antibody responses of mice immunized with diatom-expressed IbpA DR2-GFP or IbpA DR2 H:A-GFP indicate that immunization with diatom cells expressing antigens provides a promising platform to be further explored for vaccine production. A significant difference in our approach relative to standard immunizations was that we injected diatom cells, and not just purified protein, into the mice. This is based on the demonstrated improved efficacy of “particulate vaccines” over soluble proteins (O’Hagan and Singh 2014). Antibody responses were observed with whole cell and sonicated recombinant diatoms expressing IbpA DR2 H:A-GFP. In particular, treatment of mice with 5 μg of diatom-expressed IbpA DR2 H:A-GFP (w) without additional adjuvant (Fig. 8) stimulated a response relative to controls in two out of four mice. One contribution to the response could be that the diatom silica functions as an adjuvant, which we demonstrated in Newcastle disease virus experiments in chickens (Nazmi et al. 2016). With the *E. coli*-expressed IbpA rDR2, mice were exposed to a single purified protein, whereas in the diatom samples, expressed protein was present only at around 1% in a milieu of other native diatom proteins. Since the other proteins could also elicit an immune response, the sensitivity of the system to specifically respond to IbpA DR2 H:A-GFP could be compromised, and yet, a response was observed. Essentially no differences were observed comparing sonicated with whole cells, suggesting that under the conditions used, IbpA DR2 H:A-GFP was similarly available. Statistics are limited with only four treatment animals, and there are other variables to consider in this process; however, several concepts about the diatom system relate to established principles in stimulation of the vaccine-induced immune response. One could speculate that diatom cells would experience a higher residence time in mice relative to an injected protein and function analogously to a sustained release/particulate vaccine, which has demonstrated positive effects on the immune response (Gordon et al. 2012; O’Hagan and Singh 2014). Particulate carriers can significantly enhance the immunostimulatory action of antigens by having similar dimensions as pathogens the immune system is trained to recognize, and protection within a particle enables prolonged antigen exposure time (Gamvrellis et al. 2004; Liang et al. 2006). Delivery of particle-associated antigen has been shown to be substantially more effective than delivery of a soluble antigen (Edelman 2003; Foged et al. 2005). A more thorough study will be required to provide a clear evaluation of the relationship between accessibility of the desired antigen expressed in diatoms and the immune response, but the fact that we observe a response with diatom-expressed IbpA DR2 encourages pursuing such an investigation.

Both diatom-expressed IbpA DR2-GFP and IbpA DR2 H:A-GFP induced antibody responses, with the H:A mutant performing a little better in particular mice (Fig. 8). We previously demonstrated that IbpA rDR2 protects mice and cattle against experimental *H. somni* septicemia and pneumonia, respectively (Geertsema et al. 2008; Geertsema et al. 2011). Induction of a response in mice using diatom IbpA DR2-GFP and IbpA DR2 H:A-GFP provides hope that the non-toxic mutant antigen may be protective in vivo. Improving immune responses and testing protection in challenge models are the focus of ongoing research.

## Electronic supplementary material

ESM 1 (PDF 303 kb)
