# Supplementary material for: Expression of Histophilus somni IbpA DR2 protective antigen in the diatom Thalassiosira pseudonana
Source: Appl Microbiol Biotechnol. 2017 Apr 12;101(13):5313–24. doi: 10.1007/s00253-017-8267-8 (PMC5486823; doi:10.1007/s00253-017-8267-8)
Supplement: Supplementary file 1 — (PDF 303 kb) [file 253_2017_8267_MOESM1_ESM.pdf]

Supplementary Material

Journal of Applied Microbiology and Biotechnology

Expression of *Histophilus somni* IbpA DR2 protective antigen in the diatom *Thalassiosira pseudonana*

Aubrey Davis[1], Lauren T. Crum[2], Lynette B. Corbeil[2], Mark Hildebrand[1**]

[1] Marine Biology Research Division, Scripps Institution of Oceanography, UC San Diego, La Jolla, CA

[2] Department of Pathology, School of Medicine , UC San Diego, San Diego, CA

**Corresponding author, mhildebrand@ucsd.edu, phone: 858-822-0167, fax: 858-534-7313, 9500 Gilman Drive, La Jolla, CA 92093-0202

Supplementary Figure S1 Construct maps. Design of constructs used to generate transgenic diatom lines. Uninformative regions of construct maps have been omitted for clarity.

Supplementary Figure S2 IbpA DR2 sequences. a) Codon optimized and mutagenized IbpA DR2 nucleotide sequences in FASTA format. Altered nucleotides underlined and bold. b) Amino acid alignment showing the H:A mutation in bold

Supplementary Table S1 Primers used for amplification and construct synthesis

Figure S1

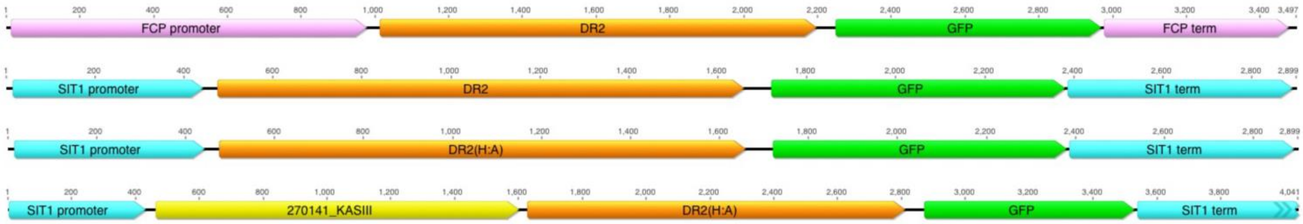
FCP promoter
DR2
GFP
FCP term
SIT1 promoter
DR2
GFP
SIT1 term
SIT1 promoter
DR2(H:A)
GFP
SIT1 term
SIT1 promoter
270141_KASIII
DR2(H:A)
GFP
SIT1 term

Figure S2

a) Nucleotide Sequences:

>DR2_Domain_Codon_optimized_for_Tp_Start_Met_added
ATGTCTGTGAACGAGGTGGCTCCACTCTCTTCTGTGCCAGCTGAGCTCAAGGATGCTGCTGGAGGAAACAA
GAAGGCTGCTGAGAAGTCTGAGGGAGCTACCGGAGTGGAGAAGGAGAAGACCACCCTCTTCCAACGTGTGA
AGCAATTCTTCACCGGATCTAAGTCTGGAGCTAAGCCAGTGGCTGGAGATGAGACCGCTAACAAGGTGAAC
TACCAAGATCTCGAGGATAACCTCAACCTCAAGGGACTCATCTCTCTCGAGGATGATCGTAACGCTAACTT
CGAGTCTAACGTGCTCAAGAACGAGAAGTTCCTCGATGAGGCTCGTGAGATCTCTAAGAAGTCTATCCCAG
AGGCTACCGTGAAGCAAATGTCTCACCTCCCAGAGTTCGATGATATCCTCACCGAGGGAGCTAAGAAGGTG
GAGTCTCGTATCAACAAGGCTATCACCTTCCGTCCATCTGTGGAGGAGTTCTCTGAGATCCAAGATCTCGT
GAAGACCCTCCCAAAGACCAAGGTGATCGAGGATCTCTCTACCAAGACCAACGAGATCACCGAGGCTCTCG
CTGCTACCTCTAAGACCATCCAACGTACCCCAGAGCTCAAGGAGCAACTCAAGACCGCTATCGAGGATTTC
CTCCAAAACTCTCAAGGAAAGCCACTCACCGTGCAAATGATCGAGAACCTCAACCACGGACTCCGTCCAGA
TGAGGGAGAGGGACGTCTCCTCTACAAGAAGGAGAACCTCACCAAGGAGAACGCTGTGTTCTCTTCTCCAG
AGGCTGCTAAGATCCAACTCGCTGAGACCGTGGATTTCATCAACCGTGCTAAGAACGAGGGAATCGAGCCA
TCTGTGGTGGGAGCTCTCGTGTACCAACGTCTCATCGCTTACCACCCATTCGCTGAGGGAAACGGACGTAT
GGCTCGTGTGATCGTGAACAAGATCCTCCTCGATGCTGGATACCCAGCTTTCACCAAGTTCTCTGATGAGT
TCGAGCCACAAATCATCCCACAAACCAAGGCTTCTACCAAGTCTGCTACCTCTTCTGAGGTGGTGGTGGAG
TTCCTCAAGGAGCTCGCTAAGAAGGGATCTAAGGAGGATAACGAGCAAAACCTCGAGAAGACCGATCGTAC
CTCTACCGATCTCACCGAGTCTGCTGTGGAGAACTCTGCTGCTCTCTCT

>DR2_Domain_Codon_optimized_for_Tp_Start_Met_added_H:A_mutant
ATGTCTGTGAACGAGGTGGCTCCACTCTCTTCTGTGCCAGCTGAGCTCAAGGATGCTGCTGGAGGAAACAA
GAAGGCTGCTGAGAAGTCTGAGGGAGCTACCGGAGTGGAGAAGGAGAAGACCACCCTCTTCCAACGTGTGA
AGCAATTCTTCACCGGATCTAAGTCTGGAGCTAAGCCAGTGGCTGGAGATGAGACCGCTAACAAGGTGAAC
TACCAAGATCTCGAGGATAACCTCAACCTCAAGGGACTCATCTCTCTCGAGGATGATCGTAACGCTAACTT
CGAGTCTAACGTGCTCAAGAACGAGAAGTTCCTCGATGAGGCTCGTGAGATCTCTAAGAAGTCTATCCCAG
AGGCTACCGTGAAGCAAATGTCTCACCTCCCAGAGTTCGATGATATCCTCACCGAGGGAGCTAAGAAGGTG
GAGTCTCGTATCAACAAGGCTATCACCTTCCGTCCATCTGTGGAGGAGTTCTCTGAGATCCAAGATCTCGT
GAAGACCCTCCCAAAGACCAAGGTGATCGAGGATCTCTCTACCAAGACCAACGAGATCACCGAGGCTCTCG
CTGCTACCTCTAAGACCATCCAACGTACCCCAGAGCTCAAGGAGCAACTCAAGACCGCTATCGAGGATTTC
CTCCAAAACTCTCAAGGAAAGCCACTCACCGTGCAAATGATCGAGAACCTCAACCACGGACTCCGTCCAGA
TGAGGGAGAGGGACGTCTCCTCTACAAGAAGGAGAACCTCACCAAGGAGAACGCTGTGTTCTCTTCTCCAG
AGGCTGCTAAGATCCAACTCGCTGAGACCGTGGATTTCATCAACCGTGCTAAGAACGAGGGAATCGAGCCA
TCTGTGGTGGGAGCTCTCGTGTACCAACGTCTCATCGCTTAC**gct**CCATTCGCTGAGGGAAACGGACGTAT
GGCTCGTGTGATCGTGAACAAGATCCTCCTCGATGCTGGATACCCAGCTTTCACCAAGTTCTCTGATGAGT
TCGAGCCACAAATCATCCCACAAACCAAGGCTTCTACCAAGTCTGCTACCTCTTCTGAGGTGGTGGTGGAG
TTCCTCAAGGAGCTCGCTAAGAAGGGATCTAAGGAGGATAACGAGCAAAACCTCGAGAAGACCGATCGTAC
CTCTACCGATCTCACCGAGTCTGCTGTGGAGAACTCTGCTGCTCTCTCT

b) Amino acid alignment of mutant vs. native IbpA DR2 domain:

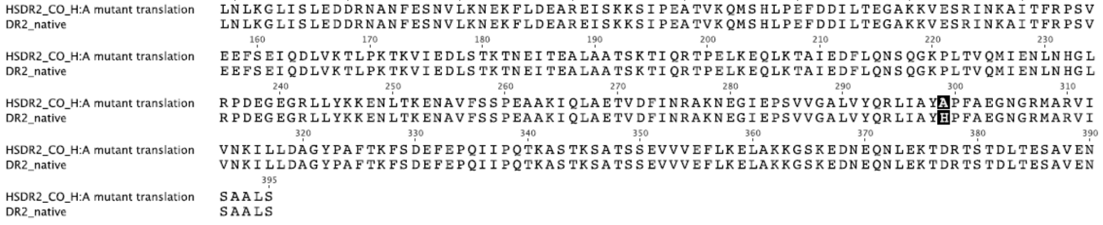

Supplementary Table S1. Primers used for amplification and construct synthesis

Gene	Construct	Forward Primer	Reverse Primer
IbpA DR2	Codon optimized gene	5'- ATGTCTGTGAACGAGGTGGCTC-3'	5'- AGAGAGAGCAGCAGAGTTCTCC -3'
Thaps3 270141	Chloroplast targeting	5'- ATGAGACTCTCCATCGCAACTATA -3'	5'- ACCCCACTTCAAGATTGCTGCAC -3'
